# Feasibility and psychophysical effects of immersive virtual reality-based mirror therapy

**DOI:** 10.1186/s12984-022-01086-4

**Published:** 2022-10-07

**Authors:** Chris Heinrich, Nadine Morkisch, Tobias Langlotz, Holger Regenbrecht, Christian Dohle

**Affiliations:** 1 Department of Information Science, Otago University, Dunedin, New Zealand; 2 MEDIAN Klinik Berlin-Kladow, Berlin, Germany; 3 Center for Stroke Research Berlin, Charité, University Medicine Berlin, Berlin, Germany; 4 P.A.N. Center for Post-Acute Neurorehabilitation Fürst Donnersmarck Stiftung, Berlin, Germany

**Keywords:** Mirror therapy, Stroke rehabilitation, Virtual reality, User study, Upper limb, Clinical feasibility

## Abstract

**Background:**

Virtual reality (VR) has been used as a technological medium to deliver mirror therapy interventions with people after stroke in numerous applications with promising results. The recent emergence of affordable, off-the-shelf head-mounted displays (like the Oculus Rift or HTC Vive) has opened the possibility for novel and cost-effective approaches for immersive mirror therapy interventions. We have developed one such system, ART-VR, which allows people after stroke to carry out a clinically-validated mirror therapy protocol in an immersive virtual environment and within a clinical setting.

**Methods:**

A case cohort of 11 people with upper limb paresis following first time stroke at an in-patient rehabilitation facility received three interventions over a one week period. Participants carried out the BeST mirror therapy protocol using our immersive VR system as an adjunct therapy to their standard rehabilitation program. Our clinical feasibility study investigated intervention outcomes, virtual reality acceptance and user experience.

**Results:**

The results show that the combination of an immersive VR system and mirror therapy protocol is feasible for clinical use. 9 out of 11 participants showed some improvement of their affected hand after the intervention. The vast majority of the participants (9/11) reported experiencing some psycho-physical effects, such as tingling or paraesthesia, in the affected limb during the intervention.

**Conclusions:**

Our findings show that immersive VR-based mirror therapy is feasible and shows effects comparable to those of conventional mirror therapy.

*Trial Registration* Trial was registered with the ISRCTN Registry (ISRCTN34011164) on December 3, 2021, retrospectively

## Background

Stroke is the second leading cause of death and the leading cause in disability of adults affecting approximately 14 million people a year worldwide [1–3]. More than half of people after stroke will suffer from a paresis in their upper limb and some form of deficit will still be present years after the stroke [4, 5].

Virtual Reality (VR) has been used for stroke rehabilitation for many years often targeting motivation, engagement and clinician control during the rehabilitation intervention [6]. A number of literature reviews have been carried out that show: VR may be beneficial as an adjunct therapy for improving upper limb function [7], VR can help drive neuroplastic change by incorporating neuroplasticity concepts of repetition, intensity, and task-oriented training of the affected limb [8], and evidence on its effectiveness is limited but encouraging [9].

There are largely two distinct categories of VR systems [10]. The first are robotic assisted systems (exoskeletons) which primarily provide stroke survivors mechanical assistance with motor training of limbs [11] and VR is often used as a visual (secondary) add on to guide survivors through a task-oriented or gameplay scenario. Reviews of these robotic systems have been carried out that show its potential benefit for survivors in terms of consistency and repeatability of the assisted movements [12, 13]. However, they remain outside the scope of this article as they attach instruments to the survivor which we aim to avoid. Also they provide assisted movements to survivors (with those attached instruments) whilst we focus on the manual movement carried out by survivors without any assisted technology. This leads us to the second type of VR systems where VR itself is the primary driving force for the therapeutic intervention. VR systems can be separated into three categories based on their level of immersion: non-immersive, semi-immersive and immersive [14, 15]. Ma and Zhen (2011) define these three categories based on their ability to isolate the user from foreign stimuli not present in the virtual environment (ie. their real environment) [14]. Non-immersive VR is defined as when a user is placed in a virtual 3D environment that can be manipulated by a conventional graphics workstation by using a monitor, a keyboard and a mouse. Semi-Immersive VR is defined as when a user is presented with a wide field of view of virtual content by either a large computer monitor, large screen projector or multiple television projector systems. Immersive VR is defined as when the user wears a head-mounted display (HMD) so that the user’s field of view is completely surrounded by virtual content. Fully immersive VR can offer people with stroke benefits [16] such as : (1) being completely immersed in the virtual illusion which can lead to more convincing “fooling of the brain” because of the mixing of what is real (hand movements, real-world/virtual environment one to one correspondence/calibration) and augmented (mirrored hand position and movement). (2) Allows for the mirrored virtual hand to be observed in the most spatially congruent and natural position for the person with stroke. (3) It disconnects the person with stroke from their real clinical environment, which often consists of distracting stimuli and allows them to focus their complete attention on their rehabilitation exercises.

Mirror Therapy (MT) was originally developed by Ramachandran in 1994 and has been used to treat many neurological impairments [17, 18]. After the first mention by Altschuler and colleagues [19], the use of MT to improve motor function in post-stroke upper limb paresis increased and several high-quality trials were published [20]. In the original execution of MT, hereinafter referred to as conventional MT, a person sits at a table on which a mirror is placed on the midsagittal plane. Movements of the unaffected limb are observed in the mirror. At the same time the affected limb is placed behind the mirror [18]. A recent Cochrane meta-analysis review was conducted that examined MT on hemiparesis after stroke and its effectiveness for improving motor function as well as other aspects (motor impairment, activities of daily living, pain) [20, 21]. They found moderate quality evidence that MT has a significant positive effect on motor function and motor impairment as adjunct therapy.

While carrying out conventional MT, a sensation can be experienced in the affected limb that is often referred to as a tingling or paraesthesia [22–24]. These perceptions ranged from a tingling sensation to a minimal involuntary movement in the hand. Video mediated MT which flickers between non-mirrored and mirrored real time images of the person’s non-affected limb at regular intervals showed that approximately 50% of observers experienced some form of these effects [22]. Weber and colleagues [25] reported of MT in a VR set up that many demonstrated involuntary movements that mirrored the actions of the unaffected limb.

Immersive VR systems have been developed to carry out mirror therapy with differing setups and rehabilitation protocols [25–31], including two described systems papers without any study results [27, 28]. Weber and colleagues [25] presented a system which uses the Oculus Rift and its accompanying hand controllers to carry out mirror therapy. The person with stroke held one hand controller and experienced one virtual mirrored hand in the immersive environment. Their described protocol consisted of 12 sessions which were made up of three 5 min segments where 11 people with chronic stroke carried out the one handed, mirrored therapy using the hand controller for movement/interaction. The three segments consisted of different tasks including: Exercise (10 repetitions of different seven exercises including wrist flexion/extension, forearm pronation/supination, amongst others), Rock Stacking (stack virtual rocks), and Functional Task (five functional tasks including stacking plates, moving fruit from one to another, amongst others). Two of the three segments (2 & 3) involved interacting with virtual objects as part of the intervention. Their pilot study results indicated that the immersive VR system and protocol was safe/well tolerated (adverse event tracking, simulator sickness questionnaire, and adherence) and that a small improvement was detected with the FMA-UE assessment (although this difference was not significant).

Mekbib and colleagues [31] developed a system which uses the HTC Vive and Leap Motion to provide mirror therapy while also allowing a therapist to monitor/assist the person with stroke while performing the rehabilitation hand exercises. Their system allows for unilateral (one limb shown) and bilateral (both virtual limbs shown) interaction modes with both mirroring/non-mirroring options available for the therapist to use with their patient. They ran a study comparing VR MT (12 people with subacute stroke) and occupational therapy (13 people with subacute stroke) which also involved fMRI imaging before and after the intervention. Their described VR rehabilitation protocol consisted of participants carrying out reaching, grasping, and releasing tasks (involving virtual objects) for 1 h a day, 4 days a week for 2 weeks. This protocol involved patients being instructed to grasp and release a target virtual ball into a basket in the virtual environment. The number of limbs shown (unilateral/bilateral) and mirroring conditions (mirroring/non-mirrored) for each patient was determined by the therapist based on the patients interest and motor capability each session. Their results showed that the VR MT group significantly improved in their FMA-UE assessment (compared to OT group). The fMRI imaging showed that neural activity increased in the brain areas implicating mirror neurons. This and the previous study [25] are using objects within the user’s view, which could be argued would distract from the user focusing fully on the mirror therapy illusion.

Lin and colleagues [29] developed an immersive VR system to carry out mirror therapy using an Oculus Rift and Leap Motion hand tracking camera. Their developed system presented people with stroke two virtual capsule (skeleton-like) hands with the unaffected hand controlling both virtual hands (one is unmirrored, the other mirrored). The hand tracking camera was placed on the table and the person with stroke held their hand in front of them, above the camera to carry out their protocol. The described protocol consisted of 7 hand rehabilitation activities which were repeated 50 times each for 30 min. Their study consisted of young healthy participants and people with chronic stroke. These two groups of people were then split into either conventional mirror therapy or VR mirror therapy. Focusing on the results of the people with stroke who carried out VR mirror therapy (9 participants), their results showed that significance was detected in the FMA-total scores and FMA-hand subset scores amongst this VR MT group of people with chronic stroke (from the pre and post-assessments).

Hsu and colleagues [26] used the same system described previously [29] to run a randomized controlled trial consisting of three groups: conventional occupational therapy, mirror therapy and VR-based mirror therapy. The VR-based MT group was made up of 18 people with chronic stroke. The VR-based mirror therapy protocol consisted of six different upper limb rehabilitation movements (including finger extension/flexion, wrist extension/flexion, amongst others) which were repeated 50 times each over 30 min. Their results show that a significant difference was detected for the FMA-UE (total score) between MT and VR MT groups from both baseline to post-assessment and baseline to 12 week follow up assessment.

These previously mentioned immersive VR mirror therapy systems/studies follow a theme often found in this area of research: promising results in regards to certain aspects, limited sample sizes, systems which all carry out mirror therapy differently, and rehabilitation protocols which were developed specifically for the VR system (i.e. not an established rehabilitation protocol). In this work, we developed an immersive VR system built upon an established, used in-daily practice mirror therapy protocol. By doing so, we have justification for our work on (1) how to carry out mirror therapy and (2) a well-developed/mature mirror therapy protocol which is used in daily practice at rehabilitation clinics in Germany. In this work, we are investigating whether immersive VR can be used to carry out an established mirror therapy protocol, which we argue would represent clinical feasibility.

A structured, standardized guideline that details how MT should be carried out by people with an upper limb paresis as they undergo their conventional MT, is provided by the Berliner SpiegelTherapieprotokoll (BeST) [32, 33]. This protocol provides a standardization in performance and documentation of the MT process and is used in daily practice at rehabilitation clinics in Germany. Morkisch and colleagues [33] indicate on the basis of a meta-analysis that the effects to improve motor function and motor impairment of the affected limb depend on the therapy protocol. A unilateral movement execution with observation of these body movements offers an advantage over bilateral movements (in front of and behind the mirror) or with the manipulation of objects. The BeST protocol contains basic hand movement classes as well as different so-called modifications. These up to 125 different combinations of movements provide the person with both a clear pathway to progress and variety in the different hand/arm exercises to keep the rehabilitation engaging even it is repetitive.

Recently, the BeST protocol has been used with a non-immersive augmented reality system called Augmented Reflection Technology (ART) [34, 35]. The person’s hands were captured by a web camera behind the monitor. This setup allowed for suitable ergonomics and visual manipulation for the user. The BeST protocol was adapted for virtual reality environments and subsequently named the BeST-ART protocol [35]. This protocol was adjusted for the inherent changes that occur when going from a real physical mirror (BeST) to a virtual mirror (BeST-ART). The biggest change was having the hands captured from above with a web cam and how this limits being able to view certain BeST hand exercises correctly. Two clinical studies have been run using this system at different rehabilitation facilities which featured people in the sub-acute phase after stroke [35]. The results of these studies showed that users were able to carry out the BeST-ART protocol within their non-immersive virtual environment. Another finding was that when used as an adjunct therapy, people after stroke were able to meet the required number of sessions as dictated by the protocol. The associated usability results showed that therapists and users both rated the system highly effective and efficient as well as wanting to continue to use the system in the future.

As a next step, as visual distractions from the real environment could disturb a person’s attention/gaze to the mirrored hand illusion, an immersive VR rehabilitation system was developed. The foundational work for this has been carried out with the development of an immersive mixed-reality system that measures aspects of embodiment for mirrored/non-mirrored personalised virtual hands amongst healthy users [36]. This study focused on embodiment as an enabling component for therapeutic efficacy. The sense of embodiment can be broken into three sub components: the sense of self-location, sense of agency, and sense of ownership [37]. These components of embodiment can be thought of as the requirements needed for MT in relation to the mirrored hand (i.e. the user needs to feel like the mirrored hand moves like their hand and they take ownership of that virtual hand). The developed system uses a Leap Motion hand tracking camera^1^, placed on the front of a head-mounted display (HMD) and has the user interact in a serious game with two conditions (mirrored/non-mirrored) and also with different visualized virtual hands and skin textures. Importantly, they only present the user with one virtual hand during their use with the system as that same requirement is necessary for MT. Results from that study show that healthy users were able to achieve an overall sense of embodiment in their virtual mirrored hand. Results from an associated second study that examined personalised virtual hand size and its effect towards perceived embodiment could not detect any difference between a default sized virtual hand and a personal sized virtual hand (mirrored or non-mirrored). Since this work provokes the intended MT effect in an immersive VR environment, we used this system as a blueprint and adapted the VR system for clinical use [36]. We combined that system with an adapted BeST-ART protocol for immersive VR use to run the clinical study evaluating its feasibility, acceptability, tolerability and clinical efficacy.

In this article, we present our developed immersive VR rehabilitation system that allows people after stroke to carry out a validated MT protocol in a clinical setting. Instead of using a tailor-made rehabilitation protocol to show the effects of VR for mirror therapy, we used an established mirror therapy protocol (BeST) and built an immersive VR mirror therapy system to support that therapeutic protocol. Hence, our main novelty and contribution lies in the ecological approach of our study demonstrating actual feasibility. We investigated its clinical feasibility with a user study at an inpatient rehabilitation clinic. These findings can be used as the basis for a larger trial. We also explored detecting any adverse effects and people’s feelings towards acceptance of the technology in general. Finally, we discuss psycho-physical effects that were observed and reported by the users and therapists during the execution of the intervention in the immersive virtual environment.

## Methods

**Fig. 1:**
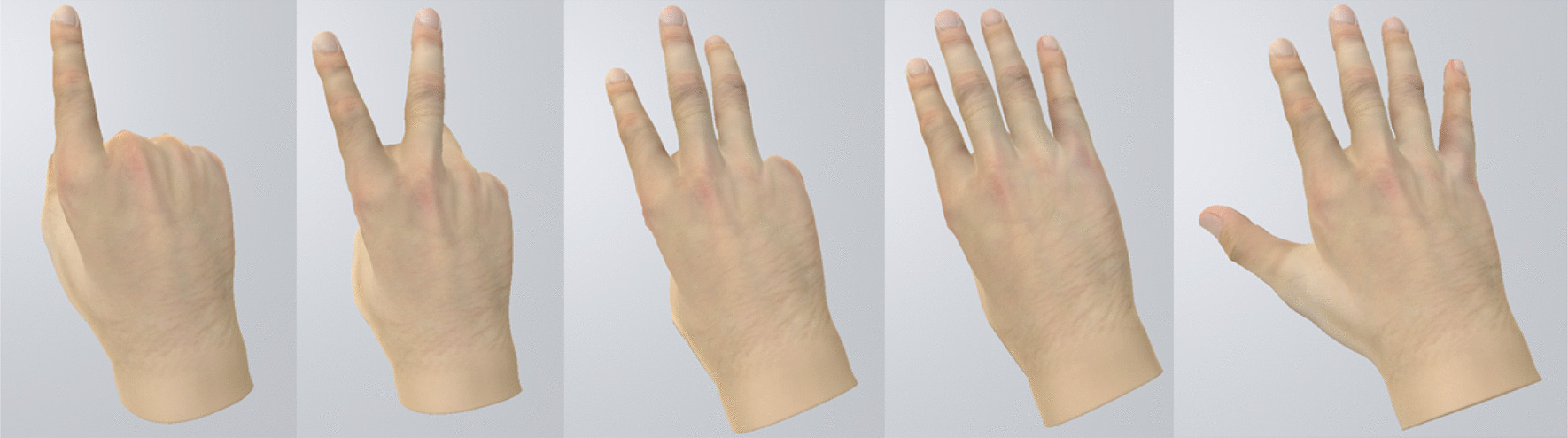
People after stroke were asked to carry out a subset of the BeST-ART protocol with the unaffected hand while using the ART VR system. These hand exercises consisted of showing the numbers 1–5 with different modifications added (palm up/down, wrist extensions, arm extensions)

To allow people after stroke to carry out MT in an immersive virtual environment, we adapted the BeST-ART protocol for immersive VR usage and adapted the ART VR setup to meet clinic requirements (Fig. 1).

### BeST-ART VR protocol

The BeST protocol was designed and validated for carrying out MT with a physical mirror [32, 38]. It requires the presentation of well-defined body postures in response to verbal commands, consisting of so-called basic movements and modifications that can be combined in order to control for complexity. The adapted BeST protocol for non-immersive VR rehabilitation, called BeST-ART protocol was modified for the VR system [35]. The same procedure, but a subset limited to 30 of those defined body positions, called as BeST-ART VR protocol, has been chosen to be carried out with our system. The BeST-ART VR protocol included one of the three basic movements (Numbers) because of hardware constraints from the hand tracking camera. The Numbers basic movement was decided upon because all hand exercises (numbers 1–5) could be captured and shown correctly.

All modifications from the original BeST protocol were available for use. Movements of modification I include two body positions of wrist extension/flexion (a) and palm up/palm down (b). Two or three different positions of modification II include elbow extension/flexion (c), hand sliding (d). The therapists used the original documentation sheet of the BeST protocol to record persons’ time spending with the system and to judge e.g. attention, level of difficulty or reporting of occurred paraesthesia.

### Clinical ART VR system

Compared to the original ART system [39], the ART VR system^2^ [36] was adapted for clinical settings by making a number of modifications to fit the new clinical environment. The Leap Motion camera was moved from being attached to the front of the HMD to being placed statically in the environment. This was done to remove a “swimming” hands effect that users can experience when the user’s hands remain stationary but they move their head. The slight delay before the camera can correct the virtual hands position according to the movement can produce a swimming like effect of the virtual hands (this is an inherent problem with that technology).

The Leap Motion camera was, therefore, placed so that it would never move during the experience and would not produce any of these possible effects. To meet the hygienic standards required in a clinical setting, disposal sanitary VR masks were incorporated with use of the HMD and spray disinfectant was used on the table top before and after each use. A height adjustable table was used to allow for users who might need the table moved up or down for comfort or accessibility (e.g. wheel chair). An arm rest with a clamp was added to allow for the user to rest their affected elbow during the rehabilitation exercises.

**Fig. 2:**
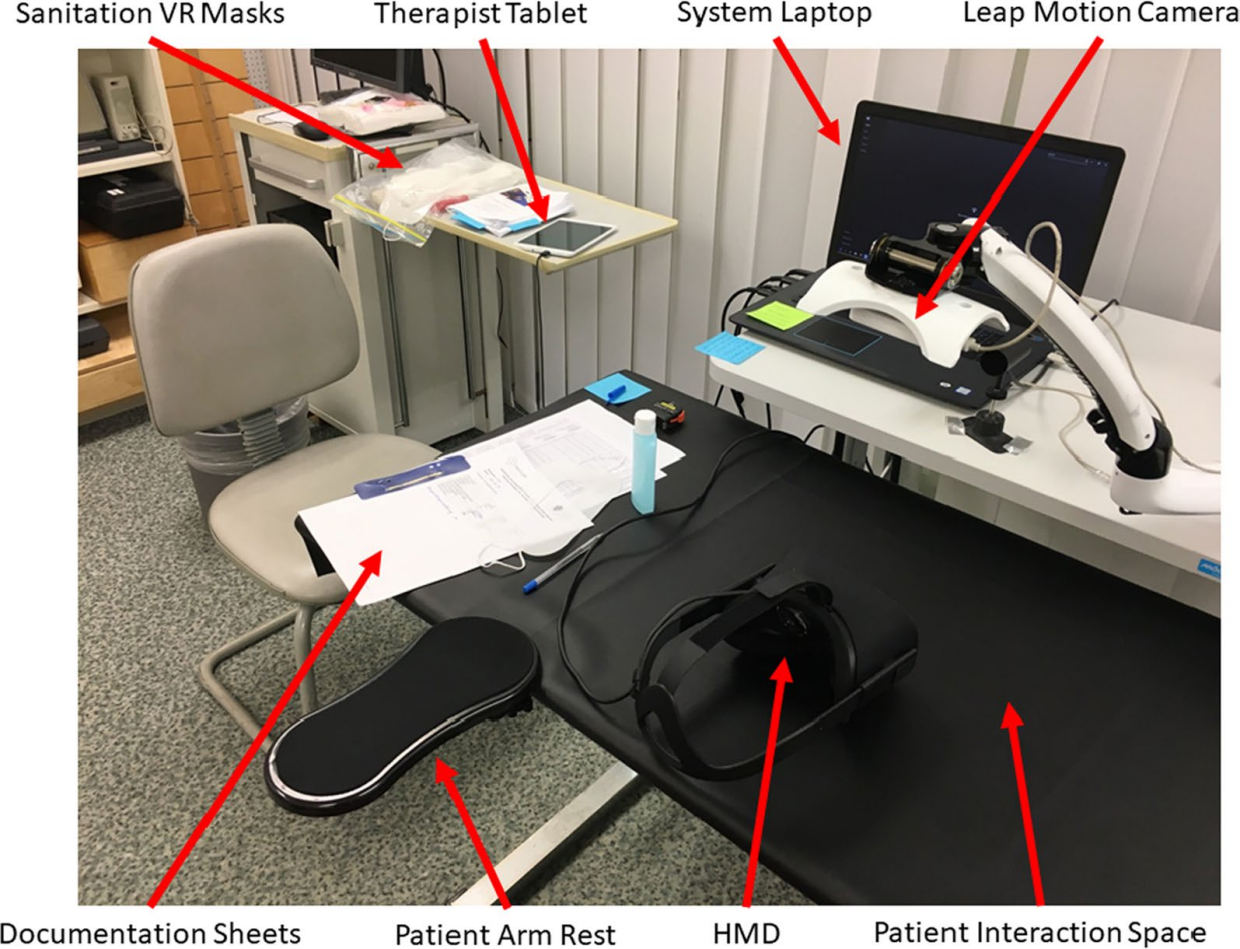
The ART VR system set up in a clinical setting which placed an emphasis on the user’s needs like a height-adjustable table, arm rest, and sanitation vr masks (and spray for the table)

#### Hardware

The clinical ART system is run on a 17inch laptop computer (Dell G3 17) which consists of a display screen (1920 × 1080 @ 60Hz), 16GB RAM, NVIDIA GeForce GTX 1060 6GB DDR5, and a Intel Core i7-8750H CPU. A Leap Motion depth sensing camera is attached to an adapted computer monitor desk mount (Digitech Desk Mount Articulating Arm CW-2870) which is angled downwards (towards the desk) and attached to an height adjustable desk. Black infrared absorbent cloth is placed on the desk for optimal leap motion tracking conditions. The head-mounted display that the person after stroke uses to experience the immersive virtual rehabilitation scene is an Oculus CV1. The Oculus tracking camera is attached to the desk (directly in front of where the user would sit) and is aimed straight towards them. The therapist controls the system via a tablet computer (Samsung Galaxy Tab A 8.0) which consists of an 8inch 1024px x 768px resolution screen, 16GB RAM, 1.2GHz Qualcomm APQ 8016 CPU and EG ULP GeForce GPU.

#### Software

Our system was built in Unity3D (v2017.3.0f3) on a Windows 10 Enterprise 64bit (1703) operating system. The Leap Motion uses the Orion SDK (4.0.0 + 52173). The Oculus application (1.38.0.261475) and HMD firmware (709) were kept consistent throughout the implementation. The therapist tablet OS is 5.0 (Lollipop) and the application is built in Android Studio (4.0) using the gradle version 3.3.0.

**Fig. 3:**
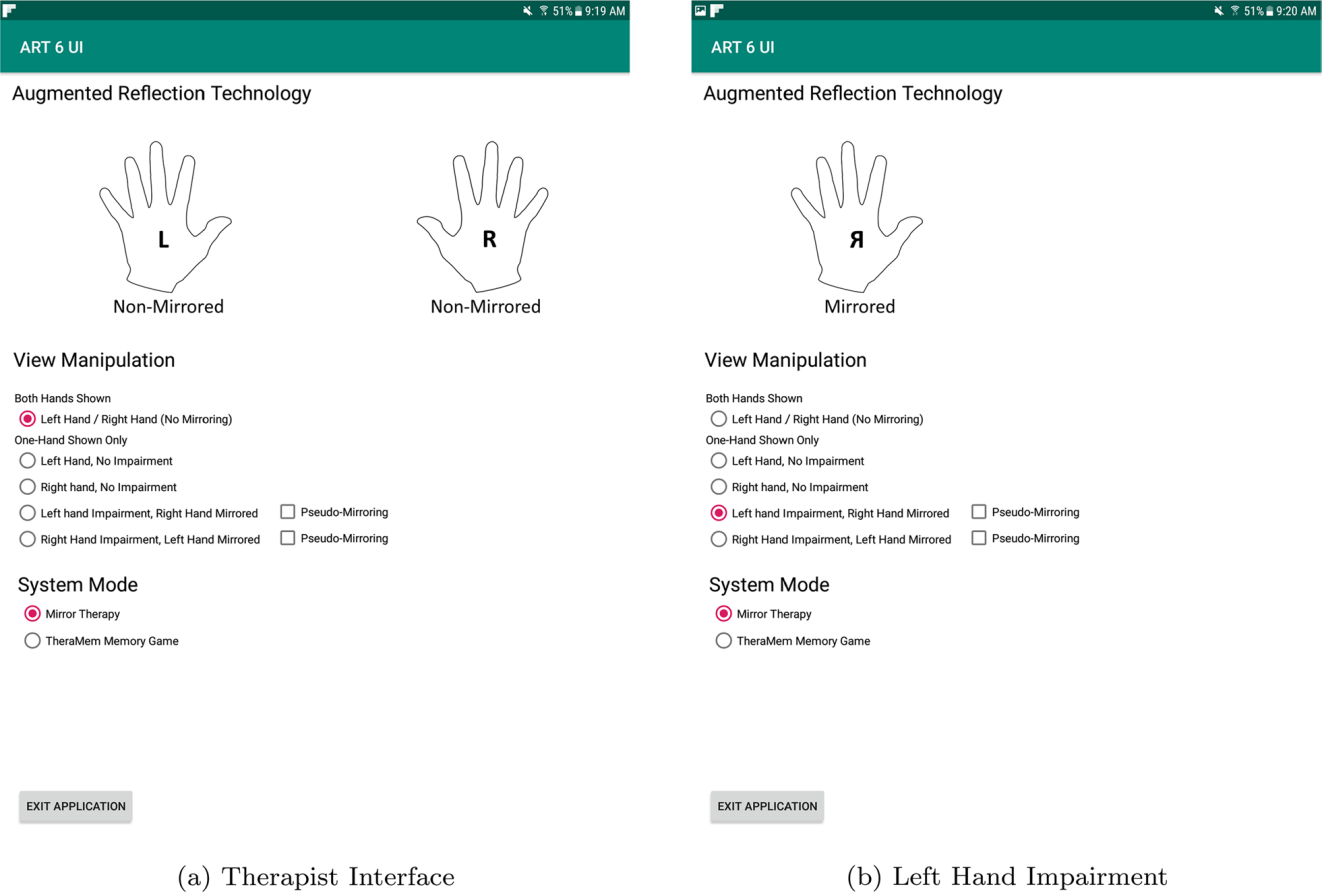
The therapist used a handheld tablet computer to control the ART VR system which allowed them to position themselves as best fit to observe the user carrying out the hand exercises

#### Therapist interface

A tablet computer was chosen for the therapists to control the VR system so that they could be free to move around and position themselves around the participant without being bound to a traditional keyboard/mouse/computer monitor interface. The procedure for the therapists to start the VR system was designed deliberately to be simple. The ART program directly opens on the system laptop (Fig. 2) into the virtual environment and the therapists have control from the therapist home screen (Fig. 3a). During this step they can select what mirroring option to use based on their participant’s affected side (Fig. 3b).

#### Virtual environment

**Fig. 4:**
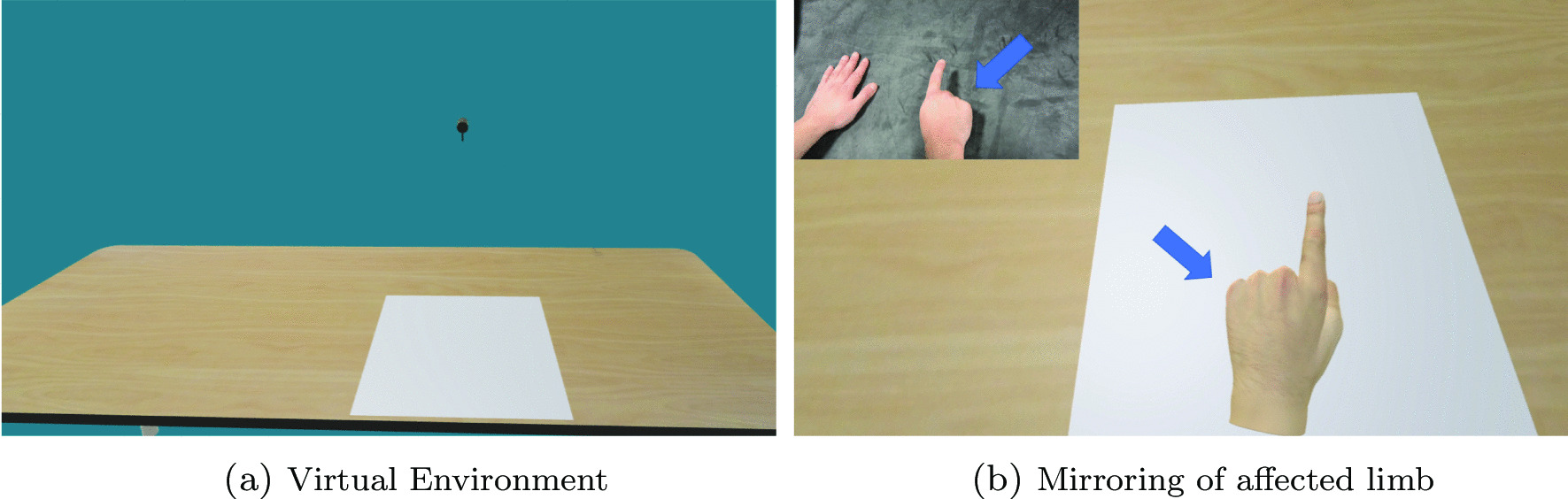
The virtual environment that the person after stroke experiences (**a**) is kept minimal and non-distracting as possible to allow the user to focus their complete attention/gaze on the mirrored hand illusion (**b**)

The BeST protocol stipulates that when people after stroke observe the mirrored hand illusion, whether on a physical mirror or computer monitor, that no objects (pens, jewellery, etc.) are present in the illusion and that there is no distracting stimulus in the background. Thus, the person focuses their complete attention and gaze on the illusion and no objects or people break the induced mirrored hand illusion. Therefore, to adhere to these BeST principles, the virtual environment the users interacted with was kept as plain as possible (Fig. 4a). The virtual environment consists of: a neutral blue-gray background, a 3D modelled wooden table, a white rectangular plane and a virtual Oculus tracking camera. The virtual and real environments (Fig. 2) were designed and calibrated to have a 1:1 distance correspondence such that when the user moves their hand 1cm, the virtual hand moves 1cm in the virtual environment as well. The white rectangular plane is where the person is encouraged to keep their virtual hand within while carrying out the defined movements for optimal Leap Motion tracking conditions.

### Clinical feasibility study

**Fig. 5:**
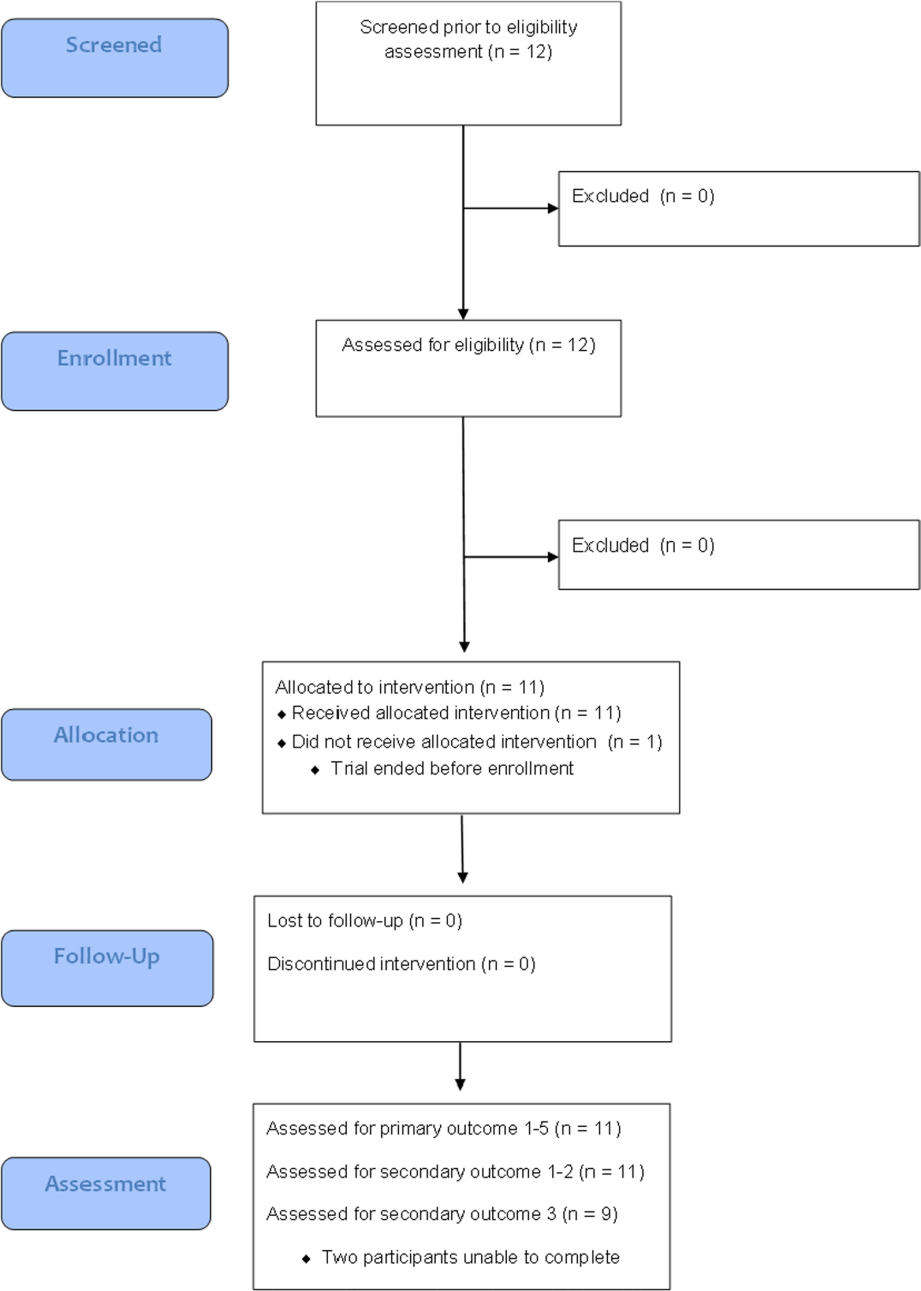
CONSORT diagram detailing the participant flow in our clinical feasibility pilot study

#### Participants

Participants were recruited at an in-patient rehabilitation facility in Berlin, Germany over a five month period. The recruitment period began in September 2019 and ended in January 2020. During the study, the participants continued to receive their therapies such as physiotherapy, occupational therapy or speech therapy. An occupational therapist screened potential participants with the following inclusion criteria: (1) at least 18 years old, (2) suffered a first-time stroke, and (3) has a severe, one-sided paresis of the hand. Exclusion criteria were defined as: (1) severe cognitive or emotional limitations, (2) insufficient sitting stability, (3) severe speech comprehension or speech production deficit, and (4) simultaneous participation in another study. All participants gave written and informed consent to take part in the study. The study was registered and approved by the ethics committee of the Charité, University Medicine Berlin, Germany (EA1/195/19). Two occupational therapists were involved with the study.

A sample size of 12 was determined from the closely related work by Hoermann et al. [35] who ran a similar clinical feasibility study which evaluated a previous version of the augmented reflection technology system.

#### Intervention protocol

All participants were seen five times, first time for pre-assessment/introduction session, three times for intervention and a final time for post-assessment. Clinical assessments were performed by the therapist before the first intervention and after the last intervention.

At least 24 h in advance to the introduction session, the potential subjects were informed by the study staff. Then they had time to decide whether or not to participate in the study and to sign their consent accordingly. The introduction session consisted of demonstrating the VR system to the person, informing them about the details of the study and what would be asked of them. Pre and post clinical assessments were conducted by the therapists. As the intervention exercises were based on a modified version of the BeST protocol (BeST-ART VR), as described below, it was recorded on the standardized BeST documentation sheets. Before and after each intervention, the therapists would sanitize the interaction space with an anti-bacterial spray. Each participant was given a sanitation VR mask for use during the intervention.

Before the start of the intervention, the therapist reminded the participants that they could withdraw from the study at any time. For all interventions, the participants started with their hand in the palm down orientation. The therapist was sitting next to the person using the tablet interface to control the system. The therapist would follow the BeST-ART VR protocol by asking the user to perform different numbers with their mirrored virtual hand. The therapist was free to use their discretion to increase or decrease the level of difficulty by changing the pace or asking them to perform different additional modifications to the basic movement (Numbers) hand exercises. Each therapy session was for 30 min in total, but this included welcoming the person, getting them seated correctly and adjusting the HMD to their head. After each instruction phase, the therapist would give the participant an individual break time. During rest periods, the participant was asked to keep the HMD on.

### Objectives

The objectives for our feasibility study were separated between the participant’s perspective (usability) and therapist’s perspective (applicability). For the participants, the objectives were whether they could use the immersive VR system and carry out the BeST-ART VR protocol while using the system. For the therapist, the objective was whether the BeST-ART VR protocol could be applied with the person after stroke.

### Outcome measurements

Different measurements were used to investigate the participant’s experience following the prescribed protocol in the immersive system. These consisted of clinical assessments for pre- and post-assessments, therapist documentation sheets and user experience questionnaires. Data was collected by the therapist and put into an MS Excel 2016 spreadsheet for analysis. Mean values (M) and standard deviations (*SD*) were calculated for the relevant demographic data, clinical assessments and questionnaires.

#### Clinical assessments

All participants underwent a pre and post assessment. The Fugl-Meyer Assessment-Upper Extremity subset (FMA-UE) was used proportionally to examine the motor impairment of the affected wrist and hand (Part B Wrist 0–10 and Part C Hand 0–14) [40]. The Modified Rankin Scale (MRS) [41] was used to measure the degree of dependence after stroke (0 = no disability to 5 = severe disability). In order to measure muscle spasticity of affected wrist/fingers, the Modified Ashworth Scale [42] was used (MAS, 0 = no increased muscle tone to 4 = rigid flexion and extension) . The subtest eleven of the National Institutes of Health-Stroke Scale (NIH-SS) [43] was used to measure extinction or inattention of the participants (0 = no inattention to 2 = profound inattention/extinction). To get information about the degree of pain in the affected arm, the Numeric Rating Scale (NRS, 0 = no pain at all to 10 = worst pain) was used. To crosscheck participant’s statement, it was used the Verbal Rating Scale (VRS, 0 = no pain at all to 10 = worst pain). Additionally, the two-point discrimination of the upper limb was assessed by the Rivemead assessment of somatosensory performance (Subtest 05) [44].

#### Mirror therapy documentation

During each of the three intervention sessions, the attending therapist would fill out the BeST documentation form [32]. This form provides a standardized method for therapists to keep track of the participant’s ability and progress throughout the BeST protocol. Information about each session includes: Time carrying out MT, rest time, modifications performed, attention/engagement in the rehabilitation, and whether they report feeling any tingling/paresthesia during the intervention.

#### User experience questionnaires

Participants user experience carrying out the BeST-ART VR protocol using the ART VR system consisted of an adapted Safety and Acceptance of Technology questionnaire from Perez-Marcos and colleagues (2017) [45], the meCUE 2.0 [46] user experience questionnaire, and the Simulator Sickness Questionnaire [47]. The Safety and Acceptance of Technology questionnaire (Tables 3 and 4) is separated into two parts: Tolerance to VR and Adverse Event Monitoring (Table 3) and Acceptance of Technology, Motivation and Self-Evaluation (Table 4). The Tolerance to VR/Adverse Event Monitoring questionnaire was filled out after each of the participants’ three interventions. The Acceptance of Technology, Motivation and Self-Evaluation was filled out after the first and last interventions. These questionnaires were translated from English to German and were cross validated by three consultants in the rehabilitation facility that were fluent in both languages. These questions were evaluated upon using a standard Likert scale (1–7, completely disagree to completely agree) as was done in the original paper, however, additional clarification (Likert-like Scale) was provided to the participants by the therapist if there was any confusion about how to apply that scale to the associated question. Questions with additional clarification given to participants were Q1–Q2 (1 = not tired at all), Q3–Q4 (1 = very relaxed), Q6 (1 = No improvement at all), Q7 (1 = Not concentrated at all) and Q8 (1 = not in a clinic room at all). We also measured for any possible simulator sickness (cybersickness). The Simulator Sickness Questionnaire (SSQ) data analysis consisted of looking at symptom severity scores which were calculated by summing up all symptom scores for each participant. The SSQ questionnaire scale is normally from 0 to 3 (none, slight, moderate, severe), however, in our study an error was made in the printing of the scale and participants were shown a scale of 1–4 with the correct corresponding symptom classifications (none, slight, moderate, severe) for each question. For analysis, 1 was subtracted from each answer to normalize our results to the usual 0–3 scale. We followed the methodology from Alghamdi and colleagues [48] to analyze the SSQ results by calculating the mean summed scores for each intervention as well as examined individual scores. We also analysed the data by splitting the questionnaire into two symptom components: (1) nausea and (2) oculomotor [49]. Kennedy and colleagues [50] provide a categorization of mean SSQ scores where a score of less than 5 is considered “negligible symptoms”, a score between 5 and 10 is considered “minimal symptoms” and a score between 10 and 15 is considered as “significant symptoms”. The meCUE 2.0 questionnaire was filled out by both the patient and therapist after the third intervention that concluded. That questionnaire and a form for analysis is available in multiple languages including German^3^.

### Outcomes

The primary outcomes of the study investigated the applicability of the developed system in conjunction with the BeST-ART VR protocol. The secondary outcomes evaluated the user experience and effects/perceptions towards the immersive VR intervention.

Primary Outcomes

1. Adherence assessed using the BeST documentation after first, second and third intervention
2. Rehabilitation dose assessed using the BeST documentation after first, second and third intervention
3. Protocol progress assessed using the BeST documentation after first, second and third intervention
4. Safety assessed using the Simulator Sickness questionnaire and VR Adverse Event Monitoring questionnaire after first, second and third intervention

Secondary Outcomes

1. Motor Impairment of the affected hand/wrist assessed using the FMA-UE subset (Part B + C) during the pre- and post-assessments
2. Perceptions regarding immersive VR intervention by person after stroke measured using the VR Acceptance, Motivation, and Self-Evaluation questionnaire after the first and third intervention
3. Tingling/paraesthesia occurrences determined by BeST documentation during the first, second and third interventions
4. User experience measured using the meCUE 2.0 questionnaire after the third intervention

## Results

### Participants

Eleven persons fulfilled the inclusion criteria and agreed to participate in the study. All of them were able to finish the trial. Five of the participants (Table 1) had experienced conventional MT in the clinic before they were included in the trial. None of them reported suffering any adverse events during the interventions. One participant (P2) was not a native German speaker and the responses were translated by an attending family member. The participant flow through the study is shown with a CONSORT diagram (Fig. 5). The included participants were on average 62.09 years old (*SD* = 8.41) and four of them were female. Ten participants stated they are right-handed and one both-handed. In eight of the eleven participants the side of the paresis was left. Ten participants suffered an ischemic stroke and one suffered a hemorrhagic stroke (mean time since stroke = 63.36 days (*SD* = 58.22 days)). Eight patients were in the subacute phase and three patients were in the chronic phase of stroke, as classified by a cut-off of 3 months after the event. All of them wore glasses which could be worn during the procedure as well.

**Table 1:** Participant Demographics and Pre-Assessment Results

ID	Age	Gender	Days since stroke	Stroke type (Hemisphere lesion)	MRS	MAS wrist/fingers	FMA-UE B/C	Exp MT
P1	51	F	206	Ischemic—Right	4	1/1	0	No
P2	66	F	63	Ischemic—Left	4	2/0	1	Yes
P3	66	M	68	Ischemic—Left	4	4/1	4	Yes
P4	67	M	15	Ischemic—Left	4	0/0	0	No
P5	75	F	103	Haemorrhagic—Right	4	1 +/1 +	1	Yes
P6	55	M	19	Ischemic—Left	4	0/0	10	Yes
P7	68	M	55	Ischemic—Left	3	0/0	0	Yes
P8	71	M	20	Ischemic—Left	2	0/0	15	No
P9	51	F	14	Ischemic—Right	4	0/0	5	No
P10	59	M	16	Ischemic—Left	2	0/0	7	No
P11	54	M	96	Ischemic—Left	3	4/4	0	No

MRS = Modified Rankin Scale, MAS = Modified Ashworth Scale, FMA-UE = Fugl-Meyer Assessment-Upper extremity subset Part B/C, Exp MT = Experience with mirror therapy previously

### Intervention

Across all three interventions, the participants’ used the system on average 13.39 min (*SD* = 3.03) per session with a range from 6.5 min to 20 min. The average time spent using the system increased with each intervention: first intervention (11.09 min, *SD* = 2.29), second intervention (13.73 min, *SD* = 2.34), third intervention (15.36 min, *SD* = 2.73). The break/rest times of the participants was 4.32 min on average (*SD* = 1.73) per intervention across all interventions. Individual participant’s time spent carrying out MT are presented in Table 2.

**Table 2:** Amount of mirror therapy time per intervention, total time spent performing mirror therapy across all interventions, post-assessment results per participant with any difference between pre and post assessments shown in parentheses

ID	1st Int	2nd Int	3rd Int	Total MT time	FMA-wrist (B)	FMA-hand (C)	FMA-UE part B/C
P1	9.5	9	10	28.5	0	0	0
P2	13	14	15	42	1 (+ 1)	2 (+ 1)	3 (+ 2)
P3	15	17	15.5	47.5	3 (+ 1)	2	5 (+ 1)
P4	6.5	11	17	34.5	0	0	0
P5	11	14	15	40	0	2 (+ 1)	2 (+ 1)
P6	12	13	15	40	7	6 (+ 3)	13 (+ 3)
P7	12	16	20	48	0	1 (+ 1)	1 (+ 1)
P8	8	13	14	35	9	10 (+ 4)	19 (+ 4)
P9	11	12	13.5	36.5	6 (+ 2)	3 (+ 2)	9 (+ 4)
P10	13	16	14	43	7 (+ 2)	4 (+ 2)	11 (+ 4)
P11	11	16	20	47	0	1 (+ 1)	1 (+ 1)

MT = Mirror therapy, FMA-UE = Fugl-Meyer Assessment-Upper extremity subset Part B/C

To start, all participants were asked to carry out the prescribed basic movements (numbers 1–5, palm down). In all of the three interventions everyone was able to perform these basic movements. In intervention 1 nine participants were able to combine the basic movement with modification Ib (change from palm up to palm down or vice versa) and therefor get ten different movement combinations. Five of them were able to perform the more challenging combination of modification IId (hand sliding) with the basic movement numbers and modification Ib (30 movement combinations). Ten were able to perform modification Ib in intervention 2 additionally to the numbers, and four were able to also incorporate modification IId. In intervention 3, nine participants were able to carry out modification Ib and six were able to combine the numbers with modification Ib and modification IId. To hold a balance of the demand between under-stress and over-stress one participant performed modification Ic (wrist extension/flexion) alternating with Ib.

### Measurements

#### Clinical assessments

Nine out of the eleven participants showed some improvement in their motor impairment of the affected wrist/hand, assessed by the FMA-UE Part B/C. The two participants who did not show any improvement (P1, P4) also carried out the least amount of MT amongst the participants (Total MT Time: 28.5 min and 34.5 min, respectively) (Table 2). Three participants improved their abilities in daily living activities. At time of the post assessment six of them were able to walk, assessed by MRS. The MAS showed a change in four participants. In one participant the muscle resistance against passive movement increased (P4) and in three participants the muscle tone decreased (P1, P2, P5). Two participants stated to have pain in the affected limb (P2, P5) at time of NRS/VRS assessments. There was no difference between pre and post assessment [(M = 0.55 (*SD* = 1.29)].

#### VR tolerance/acceptance/motivation

The VR tolerance/adverse Event Monitoring questionnaire (Table 3) that was completed after every session showed minimal negative effects from the virtual reality intervention on the participants self-reported well being. Participant’s feeling of tiredness was very similar after the intervention to what it was rated before the intervention showing that intervention did not contribute any lasting or significant exertion on the person. Participants reported low ratings for feeling relaxed before the intervention and low ratings during the intervention. Importantly, participants reported very low ratings for any unusual pain or discomfort during the intervention. Participants reported very low ratings regarding feeling any improvement in the movement of their affected limb.

**Table 3:** Tolerance to VR Intervention (Q1–4), Adverse Event Monitoring (Q5) and Self-Evaluation (Q6) Likert Scale 1—Completely Disagree to 7—Completely Agree

Question	Session number, M (*SD*)		
	Session 1	Session 2	Session 3
Q1. Before the session, how tired do you feel?	2.55 (*1.64*)	1.73 (*1.01*)	2.27 (*1.19*)
Q2. After the session, how tired do you feel?	2.73 (*1.74*)	2.27 (*1.35*)	2.73 (*1.01*)
Q3. Before the session, how relaxed do you feel?	2.36 (*1.43*)	2.91 (*2.26*)	1.73 (*0.90*)
Q4. During the session, how relaxed did you feel?	1.91 (1.04)	2.27 (*1.49*)	2.18, (*1.54*)
Q5. During the exercises, did you feel any unusual pain (e.g. stronger) at the level of the upper limbs (arms, joints, hands) or the trunk?	1.27 (*0.65*)	1.18 (*0.60*)	1.18 (*0.60*)
Q6. After the session, do you feel any improvement of the movements (e.g., larger movements, more precise, etc)?	2.27 (*2.28*)	1.55 (*1.51*)	1.55 (*1.51*)

Likert-like Scale for Q1–Q2: 1 = Not tired at all, Q3–Q4, 1 = Very relaxed, Q6: 1 = No improvement at all. This questionnaire was completed after every session. Means (*SD*) are provided for each session

The Acceptance of Technology and Motivation questionnaire (Table 4) that was completed after the first and last intervention sessions showed generally positive ratings toward immersive VR intervention. Generally, participant’s feelings remained consistent from the first intervention to the last intervention. Participants reported that they were very concentrated on the task in both sessions. Participants indicated that they did not feel like they were in a hospital room during the first intervention, however, by the last intervention this feeling had moved more to a more neutral feeling. Participants reported a high level of agency with the mirrored virtual hand and that it was carrying out their desired movements. Participants felt a high level of comfort with the BeST ART movements that they were asked to carry out. When asked if they liked the exercises, participants responded that they liked the exercises in both interventions, however, they liked the exercises slightly more in the first intervention. Participants reported an above average rating for having the impression that they were doing rehabilitation exercises. Participants had a neutral opinion about the virtual hands appearance and did not indicate one way or another whether it should look more realistic. Participants expressed that they would like to spend more time continuing the hand exercises at the hospital and would also like the opportunity to carry out the hand exercises at home.

**Table 4:** Acceptance of Technology (Q7–13) Motivation (Q14–15). Likert Scale 1—Completely Disagree to 7—Completely Agree

Question	Session, M (*SD*)	
	First session	Last session
Q7. During the exercises, were you concentrated on the task?	5.91 (*1.58*)	6.00 (*1.27*)
Q8. During the exercises, did you have the feeling of being in the hospital room?	2.27 (*2.41*)	3.27 (*2.83*)
Q9. Did the movements of the virtual hand reflect your movements?	5.91 (*1.14*)	5.45 (*1.37*)
Q10. During the exercises, did you feel comfortable with the requested movements?	5.55 (*1.29*)	5.18 (*1.94*)
Q11. Did you like the exercises?	5.82 (*1.08*)	4.64 (*1.96*)
Q12. Did you have the impression of doing rehabilitation exercises?	4.36 (*2.66*)	4.64 (*2.38*)
Q13. Would you like the virtual hand to look more realistic?	3.45 (*2.62*)	3.64 (*2.58*)
Q14. Would you like to spend more time doing the exercises at the hospital?	5.82 (*1.40*)	5.09 (*2.26*)
Q15. Would you like to continue doing the exercises at home?	5.82 (*1.89*)	5.36 (*2.29*)

Likert-like Scale for Q7: 1 = Not concentrated at all, Q8: 1 = Not in a clinic room at all. This questionnaire was completed after the first and last session. Means (*SD*) are provided for each session

#### Simulator sickness questionnaire

The SSQ questionnaire was used after each of the three interventions. All participants were able to complete the questionnaire. For the first intervention, participants reported a mean score of 2.36 (*SD* = 2.01). For the second intervention, participants reported a mean score of 2.45 (*SD* = 2.46). For the third intervention, participants reported a mean score of 2.73 (*SD* = 2.34).

From the work of Bouchard and colleagues [49], we can split the SSQ into two symptom components (nausea and oculomotor). For the first intervention, participants reported a mean nausea total of 0.46 (*SD* = 0.66) and a mean oculomotor total of 1.91 (*SD* = 1.88). For the second intervention, participants reported a mean nausea total of 0.64 (*SD* = 0.48) and a mean oculomotor total of 1.82 (*SD* = 2.55). For the third intervention, participants reported a mean nausea total of 0.55 (*SD* = 0.66) and a mean oculomotor total of 2.18 (*SD* = 2.29). According to categorization of SSQ scores by Kennedy et al. [47], these would all fall under the category of “negligible symptoms” for both nausea and oculomotor symptom components.

In terms of each individual factor of the SSQ questionnaire these five sub components had the highest mean average across all interventions: Difficulty Concentrating (M = 0.55, *SD* = 0.74), Strained Eyes (M = 0.42, *SD* = 0.74), Difficulty Focusing (M = 0.36, *SD* = 0.69), Sweating (M = 0.33, *SD* = 0.47) and Fatigue (M = 0.27, *SD* = 0.51). 6 sub components had scores of 0 across all interventions for all participants: Headache, Nausea, Dizzy (eyes open), Dizzy (eyes closed), Vertigo and Stomach Awareness.

#### meCUE

Two participants were not able to finish that questionnaire due to a language barrier and reduced capacity. The other nine participants successfully completed the entire questionnaire (Table 5). Both participants and therapists reported high ratings for module I (usefulness and usability). Module II, again had similar ratings for participants and therapist ratings with high ratings reported for visual aesthetics and low ratings for status and commitment. Module III (positive and negative emotions) reported low ratings for negative emotions for both groups. Therapist and participant ratings were both slightly below the midpoint for positive emotions. Module IV showed participants ratings slightly above the midpoint for intention to use and slightly below the midpoint for product loyalty. Conversely, therapists ratings were high for both subscales. Finally, overall evaluation was rated highly by both groups.

**Table 5:** meCUE 2.0 user experience questionnaire results for the user and therapist

Module	Subscale	Participant rating *M* (*SD*)	Therapist rating *M* (*SD*)
Module I	Usefulness	5.59 (1.00)	6.64 (0.46)
	Usability	6.26 (1.00)	6.31 (0.32)
Module II	Visual aesthetics	4.81 (1.28)	5.00 (0.49)
	Status	3.22 (1.72)	3.75 (0.51)
	Commitment	2.07 (0.95)	1.45 (0.93)
Module III	Positive emotions	3.17 (1.01)	3.97 (0.85)
	Negative emotions	2.61 (1.12)	1.67 (0.64)
Module IV	Intention to use	4.30 (1.38)	5.82 (0.46)
	Product Loyalty	3.19 (1.14)	5.88 (0.31)
Module V	Overall evaluation	3.10 (1.7)	4.20 (0.60)

Likert Scale 1 (Strongly Disagree) to 7 (Strongly Agree) for all modules except Module V (Overall Evaluation) which is from − 5 to 5. Means (*SD*) are provided for each module

#### Psychophysical effects

Participants reported a high occurrence of tingling or paraesthesia in their affected hand/arm during the interventions (Table 6). During the first intervention, nine participants reported feeling either a tingling or paresthesia sensation in their affected limb. The therapists notes indicate that for participants who reported feeling a tingling sensation, this occurred in multiple instruction phases of the intervention. All four participants who reported feeling a paraesthesia in their affected limb, indicated experiencing different sensations, e.g. pulsating wave movements under the skin on the back of the hand, a warm feeling inside the hand, a pressure on the back of the hand, or an itch on the hand.

**Table 6:** Participants reported occurrences of tingling and paraesthesia sensations in their affected hand/arm while carrying out mirror therapy in the immersive VR system across the three interventions

ID	First intervention		Second intervention		Third intervention		Total (over 3 interventions)			Clinical assessment
	Tingling	Paraesthesia	Tingling	Paraesthesia	Tingling	Paraesthesia	Tingling	Paraesthesia	Combined	FMA-UE part B/C
1	Yes	No	No	No	Yes	No	2	0	2	0
2	Yes	No	Yes	No	Yes	Yes	3	1	4	3 (+ 2)
3	No	Yes	No	Yes	No	Yes	0	3	3	5 (+ 1)
4	No	No	No	No	No	No	0	0	0	0
5	Yes	Yes	No	Yes	Yes	No	2	3	5	2 (+ 1)
6	Yes	No	Yes	No	Yes	No	3	0	3	13 (+ 3)
7	Yes	No	Yes	Yes	No	Yes	2	2	4	1 (+ 1)
8	No	No	No	No	No	No	0	0	0	19 (+ 4)
9	No	Yes	No	Yes	No	No	0	2	2	9 (+ 4)
10	Yes	No	No	Yes	Yes	Yes	2	2	4	11 (+ 4)
11	Yes	Yes	Yes	No	Yes	No	3	1	4	1 (+ 1)

During the second intervention, eight participants reported feeling either a tingling or paraesthesia sensation in their affected limb during the intervention. The five participants who reported feeling a paraesthesia in their affected limb again experienced different sensations on their affected limb, like muscle twitching of the fingers and pressure on the back of the hand, a warm feeling inside the hand and an urge to move the affected hand, a feeling like their hand was floating in one instruction phase and a feeling that it was shaking (from the shoulder to hand) in another instruction phase, a pressure on the back of the hand and a throbbing sensation in the hand, or a stretching feeling in the hand.

During the third intervention, eight participants reported feeling either a tingling or paraesthesia sensation in their affected limb during the intervention. The four people who reported feeling a paraesthesia reported their sensations in their affected limb as: ants were crawling on the hand, feeling a muscle twitching on the back of the hand, feeling the same shaking as before (from shoulder to the hand) and an urge to move the affected hand.

Across all interventions, two participants did not report any tingling or paraesthesia sensation across all interventions. For the nine participants who experienced a tingling/paraesthesia sensation, seven experienced one type of sensation for all three interventions (two other participants experienced them in two of the interventions). On average over the three interventions, participants were slightly more likely to experience a tingling sensation (1.54 occurrences, *SD* = 1.23) rather than a paraesthesia sensation (1.18 occurrences, *SD* = 1.02).

#### ART VR system setup

The therapists reported, that the ART VR system setup (Fig. 2) was easy to use, regardless of the side of the paresis or whether a wheelchair was used. The interface of the handheld tablet computer were clearly arranged, so it was easy to control the VR system. Having feedback on the participant’s view from the screen of the system laptop was helpful in order to be able to provide appropriate information to them. For two participants, the therapists mentioned it was a little cumbersome to put on the HMD because of their small head. Basically, to wear the HMD while the intervention was ongoing, was not described as uncomfortable. It was mentioned once that the lenses were foggy because the participant was sweating. The participants stated that it was not uncomfortable during rest (M = 4.32 min, *SD* = 1.73).

## Discussion

In this paper, we have shown that it is feasible for an immersive VR system to be used in a clinical setting with an established MT protocol for our limited sample size and number of interventions. We have evaluated the system in terms of clinical assessments as well as their general feelings toward VR acceptance. We have also found that tingling and paraesthesia can occur in immersive VR and perhaps even to a greater extent.

### VR intervention

Our study showed that people after stroke were able to carry out the BeST-ART VR protocol while in the immersive VR environment. All participants finished the three intervention units and the entire trial. Participant’s well-being throughout the intervention was found to be satisfactory in terms of safety and possible side effects to VR. None of them reported suffering any adverse events during the interventions. The ART VR system setup (Fig. 2) was easy to use, regardless of the side of the paresis or whether a wheelchair was used. Participants were able to wear the HMD during the intervention and rest/breaks and did not report any discomfort for wearing the HMD for that duration. The participants indicated that being in an immersive environment made them feel less like they were in a hospital room while carrying out their therapy. It was reported that they were able to concentrate on the rehabilitation to a high degree while in the immersive virtual environment and that they felt comfortable with the requested hand exercises by the therapist (Table 4). Their responses to the Simulator Sickness Questionnaire indicated they experienced close to no nausea while carrying out the hand exercises in the immersive environment.

Participants average usage time ($\approx$ 13 min) carrying out MT increased with each intervention indicating that they were engaged with the therapy and were eager to continue progressing with the BeST-ART VR protocol. Recently trials executing this MT protocol with a physical mirror (mean $\approx$ 11 min) [38] or in a non-immersive setup (mean $\approx$ 11 min) [35] showed slightly less active intervention time. The time for needing a rest was similar to the mentioned trials [35, 38]. However, the tasks, e.g. preparation and follow-up, questionnaires, in addition to the actual intervention of 30 min, differed.

Participants were sufficiently challenged by the BeST-ART VR protocol according to their capacity as indicated by the number of different posture combinations within the BeST protocol [32, 33]. Ten of the participants reached a movement combination of ten different body positions. Moreover, six participants were able to reach twenty to thirty different movement combinations in the course of the three intervention units. As indicated by the BeST documentation, a balance between under-stress and over-stress could be maintained even though the level of difficulty was increased. The positive trend in terms of active system usage time and the increase in the level of difficulty were comparable to those in the execution of conventional and non-immersive MT [35, 38]. This engagement was also shown based on their responses to the meCUE question for intention to use (Table 5), a result similar to that Hoermann et al. [35], and to the Acceptance/Motivation questionnaire. Here, they indicated that they would like to spend more time carrying out the exercises at the hospital and also at home (Q14/15, Table 4). This sentiment of wanting to continue rehabilitation at the hospital/home was also expressed in related work previously [28, 30].

A decrease of motor impairment in the affected arm, assessed by FMA-UE Part B/C could be shown in nine participants (range 1–4 points, Table 2). Out of a total of 24 points to be achieved, three participants already had a score of $\ge$ 7 at the time of the pre-measurement (P6, P8, P10). While 9 out of 11 participants were assessed with some improvement in their affected arm, their responses to the VR Acceptance questionnaire for Q6 (“After the session, do you feel any improvement...”) indicated that they largely did not report feeling improvements after each intervention (1 = no improvement at all, 1st intervention: 2.27, 2nd intervention: 1.55, and 3rd intervention: 1.55). We hypothesize that this could be the result of not being aware of any indicators occurring during the intervention for potential improvement. It is known that motor improvements after stroke are only observed on a time scale from days to weeks, as it was the case in this study. However, the patient’s questionnaire was applied immediately after every single therapy session (asking whether they had experienced any improvements from just the previous session). Thus, it is understandable that study participants only reported minor improvements on this time scale. In order to be correlated to objective measures, further evaluation studies should specifically ask for improvements experienced during the entire intervention period. With Q4 (“During the session, how relaxed do you feel?”), participants reported a low rating across all interventions (1 = totally relaxed, 1st intervention: 1.91, 2nd intervention: 2.27, and 3rd intervention: 2.18). These responses indicate that participants were largely relaxed during the intervention, this is expected as per the BeST protocol, the therapist tries to find the right balance for the patient between under-stress and over-stress to keep them engaged with the repetitive exercises.

In the related work by Brunetti and colleagues [51], they describe people after stroke as either responders or non-responders of MT. They were described as non-responders if their FMA-Fingers score was 0. In our study, 4 participants met this definition from the initial assessment (P1, P4, P7, P11) from Table 2. Two of these non-responders were able to show some improvement and increase their FMA-Hand score from 0 to 1 (P7, P11). These participants also reported experiencing tingling/paraesthesia during their interventions (4 total combined instances for both participants). They also were the participants with the longest time carrying out MT with the system (48 min for P7 and 47 min for P11, respectively). Their wrist scores remained the same (0 for both) and so their total score for the FMA-UE was 1 for both participants. P4 and P1 (who would also be described as non-responders from the definition from [51]) did not show any improvements during the post assessment. Their total combined reported instances of tingling/paraesthesia during the interventions was 0 (P1) and 2 (P4), respectively. Their time carrying out MT was also amongst the lowest of participants with 28.5 min for P1 and 34.5 min for P4, respectively. In the study of Brunetti and colleagues [51], they also followed the BeST protocol and their intervention schedule consisted of 30 min sessions for 5 days a week over 4 weeks. However, all of their participants were in the subacute stage of stroke whereas our study included three participants (P1, P5, P11) in the chronic stage as well. In their study, the 6 non-responders did not show any improvement in their FMA-Finger scores after the intervention. In our study, two of these non-responders were able to show improvement with only three interventions. It is too low of a sample size to draw conclusions (for both studies), however, it is possible that the immersive VR environment provides a more convincing mirrored illusion than one that occurs with a conventional mirror. Participants also reported high ratings of concentration on the rehabilitation task and not feeling like they were in a hospital room so it also possible that immersive VR offers a stronger decoupling of the person from their clinical environment which can often have stimulating or distracting stimuli that can affect their focus/gaze/immersion into the mirror therapy illusion.

### Tingling/paraesthesia

Across the interventions, many of the participants expressed that they were experiencing one of a variety of psychophysical sensations during the execution of the BeST-ART VR hand movements. While other studies have reported observing similar effects, to the best of our knowledge, our study is the first which has systematically tracked the effects across the interventions (Table 6).

We could not find any patterns amongst the tingling/paraesthesia data when breaking down by intervention, sensation or participant. Two participants did not report any feelings of tingling/paraesthesia. Of the participants who reported occurrences of the sensations, they all reported these sensations for at least two (of the three) interventions. We hypothesize that these occurrences of tingling and paraesthesia could be positive indicators for people after stroke that the mirror therapy illusion is having an effect and could have benefits for their motor recovery.

### Generalisability

The results from our feasibility study can be applied to other domains besides stroke rehabilitation. Mirror therapy is also used to treat phantom limb pain and hot/cold burns and we hypothesize that our developed system could be used to treat those conditions as well (with different therapeutic protocols in place). The developed system could be further modified for other rehabilitation/application scenarios where manual, embodied interaction is required. Our usability results show that it is feasible for clinical therapeutic interventions where the person to be treated and therapist are not present in the same environment (ie. patient is in an immersive VR environment while the therapist is not). In the future, these findings could also be used for remote rehabilitation where the patient and therapist are not even in the same room and the instructions to the patient could be given via verbal instruction only (like in our presented protocol). Our VR acceptance results show that people with a stroke can and want to use immersive VR hardware. When applying non-VR specific rehabilitation protocols to immersive VR setups, the potential hindrances of VR need to be accounted for (potential for simulator sickness, safety of the user while the headset is on, sanitation of the hardware/setup, etc). Stroke survivors bear a considerable risk for developing seizures or epilepsy (more pronounced for hemorrhagic than ischemic stroke [52]). Thus, there is some concern about eliciting visually sensitive seizures when using VR setups for rehabilitation. However, the literature so far does not support this concern as long as extensive visual provocations such as bright or flashing lights are avoided [53]. Our developed system consisted of commercially available, off-the-shelf hardware with minimal modifications (3D printed camera mount, cut infrared absorbent cloth) and this type of low-cost setup was shown to be feasible in a clinical setting. We hope this can inspire other researchers/clinicians in the rehabilitation field that setups do not need to consist of expensive, highly customised hardware and setups to achieve clinical results.

### Overall feasibility

We can summarise the overall feasibility of the system and protocol by linking back to the primary and secondary outcomes defined previously. Adherence to the intervention was 100% with all participants attending all three interventions and pre/post-assessments. Rehabilitation dose was an average of 13.93 min (*SD = 3.03*) across all interventions with the time increasing with each intervention. Participants were able to progress through the protocol with all participants completing the basic movements. By the end of the intervention, 10 participants were able to incorporate additional modifications (Mod I) and, of those, 6 progressed to the furthest modifications (Mod II). There were no reports of any safety related incidences or adverse events. SSQ scores on average for all participants were reported within the lowest categorisation of “negligible symptoms”. Nine participants showed improvement with their motor impairment of the affected hand including two who would traditionally be categorised as non-MT responders.

Participants perceptions towards the immersive VR intervention were largely positive with many indicating they could concentrate on the rehabilitation task, that they were comfortable with the system/protocol and that they wanted to continue the exercises in the clinic as well as at home. Nine participants reported experiencing psycho-physical effects (tingling/paraesthesia) during the interventions. The user experience results showed high ratings for usefulness and usability for both the person with stroke and therapists.

### Study limitations

An important limitation is that five participants in our study had previous experience with MT (and likely the BeST protocol) and six did not. This would give those participants a familiarity with the hand exercises they were being asked to perform and more time of MT at all. However, they would be carrying it out in VR for the first time. As participants were in-patients at the facility, they were also undergoing conventional rehabilitation as well as our immersive MT interventions, so it is difficult to report on how much effect our system/protocol had on their rehabilitation (and clinical assessments) compared to the other rehabilitation they were undergoing at the same time.

The pre and post assessments were conducted by the same therapists who performed the MT, thus it was not blinded. The meCue ratings and results for the therapists were based on their experience with the system for each participant.

Our study also has limitations in its size and design. As this study was primarily designed to investigate feasibility and acceptance, it was not designed as a (randomised) controlled trial—which should follow. We recruited from a large rehabilitation facility over 5 months and we were only able to find 11 people with first ever stroke who fit our inclusion/exclusion criteria. This is a similar problem to many researchers in the field that it is difficult to recruit a large number of people after stroke in a clinical setting when applying very strict criteria. Further trials should apply this procedure to a broader group of patients.

### VR intervention limitations

There are known limitations of the Leap Motion hand tracking camera (and of similar depth sensing cameras). We mitigated these by using black infrared absorbent cloth on the table and by limiting hand exercises to basic movements (as these hand exercises could all be accurately captured by the Leap camera). From the therapists observations, participants did not seem affected by tracking errors and their VR acceptance responses reflected this, for Q8 (did virtual hand movements reflect your movements?) participants indicated a high degree of control over the virtual hands’ movements (5.91 average for the first intervention and 5.45 for the final intervention on a 7-point Likert scale).

### Future work

For about 30–66% of stroke patients, the arm remains without function 6 months after stroke [54, 55]. Previous data on mirror therapy indicate that these patients are especially likely to benefit from MT [56], even in the chronic phase after stroke [20]. However, effective application of MT requires several therapy sessions per week for a period of several weeks [20], with therapy content continuously being adapted to the individual patient’s ability [38]. During neurorehabilitation, therapeutic resources are limited and have to be balanced between different symptoms. Thus, even as MT in general and the BeST protocol [32] in particular is widely known and used, its application is rarely delivered in sufficient intensity [57]. The adaptive VR-ART system presented in here has the potential to allow every patient with severe arm paresis to take part in a full course of (VR-) MT. This could even be applied in an outpatient setting. A standardized protocol called BeSTEP for self training is also established [58]. Early work has been done on adapting the BeST ART VR system for home rehabilitation [59] with preliminary adherence/usability results [60] showing potential for immersive VR home rehabilitation. Implementation of this therapy regime could have a major impact on clinical improvement and even the economic burden of stroke.

Clinicians following the BeST protocol [32] have indicated that they look at the person’s eyes and gaze to help determine their visual attention and engagement during the execution of the protocol. They uses this to determine whether to increase/decrease the pace of the verbal instruction or the level of difficulty or when to give the person a break/rest. Head and eye tracking could be useful features as the person’s eye is naturally concealed by the HMD during use. It could be integrated either as a ray on the therapist laptop (which is showing what the person after stroke is seeing) as a way for them to see when the person after stroke is becoming less engaged in the therapy. Machine learning could also be used with the person’s gaze to detect when they are losing attentiveness and present it to the therapist on the tablet indicating a break is needed. Also an wearable EMG device could be used for a similar function. There has been related work to analyse the data from these devices in stroke application contexts [61, 62].

Individualised hand textures could also be added to the system to possibly increase levels of ownership. Heinrich and colleagues [36] have created a process of using images to texture the virtual hands and a similar process could be followed to give people with stroke individually textured hands when carrying out their rehabilitation. Whether it is worth the effort to individually texture hands versus provide a wide set of skin complexions to choose from is unknown. The authors of that process could not detect any difference in terms of perceived embodiment between that individually textured hand and a default textured hand meaning perhaps a similar looking hand is sufficient.

Lastly, adding additional cameras to the system setup could help capture more views of the person’s hand and allow for more hand exercises to be added from the BeST-ART protocol to the BeST-ART VR protocol. Currently the system can only capture the hand from above, but if a perpendicular camera was added then that could also capture the hand from the side and it could increase the number of BeST-ART movements that the person with stroke is able to carry out in our system. This would increase the variety of hand movements by adding more of the basic movements to the protocol as well as more modifications.

Future research should investigate immersive VR-based MT with appropriate sample sizes to also be able to make a statement about the effect in relation to the type of stroke or the time since the stroke.

## Conclusions

Our study shows the potential for immersive VR hardware used in conjunction with an adapted established MT protocol in a clinical setting with a limited sample size and number of interventions. Our feasibility study investigated clinical outcomes, virtual reality acceptance/motivation/self-evaluation and user experience. Our results show potential for enhanced motor recovery for people after stroke. In particular, many of our participants experienced many occurrences of psycho-physical effects such as tingling/paraesthesia during the interventions. Our findings have implications for researchers/clinicians wanting to incorporate immersive VR hardware into clinical rehabilitation settings.

## Data Availability

The datasets used and/or analysed during the current study are available upon request from the corresponding author upon reasonable request.

## Abbreviations

ART: Augmented reflection technology
BeST: Berliner Spiegeltherapieprotokoll
FMA-UE: Fugl-Meyer upper extremity subset
HMD: Head-mounted display
MAS: Modified Ashford Scale
MRS: Modified Rankin Scale
MT: Mirror therapy
NIH-SS: National Institutes of Health-Stroke Scale
NRS: Numeric Rating Scale
SSQ: Simulator sickness questionnaire
VR: Virtual reality
VRS: Verbal Rating Scale

## Footnotes

1 https://www.leapmotion.com

2 https://hci.otago.ac.nz/bestartvr.html

3 (http://mecue.de/english/index.html)
